# Computational Stemness and Cancer Stem Cell Markers in Oral Squamous Cell Carcinoma: A Systematic Review, Dual Meta-Analysis, and Functional Meta-Synthesis

**DOI:** 10.3390/medsci14010021

**Published:** 2025-12-31

**Authors:** Carlos M. Ardila, Eliana Pineda-Vélez, Anny M. Vivares-Builes

**Affiliations:** 1 Department of Periodontics, Saveetha Dental College and Hospitals, Saveetha Institute of Medical and Technical Sciences, Saveetha University, Chennai 600077, India; 2 Biomedical Stomatology Research Group, Basic Sciences Department, Faculty of Dentistry, Universidad de Antioquia U de A, Medellín 050010, Colombia; eliana.pineda@uam.edu.co (E.P.-V.); anny.vivares@uam.edu.co (A.M.V.-B.); 3 Faculty of Dentistry, Institución Universitaria Visión de las Américas, Medellín 050040, Colombia

**Keywords:** mouth neoplasms, carcinoma, squamous cell, neoplastic stem cells, immunohistochemistry, transcriptome

## Abstract

**Background/Objectives:** Stemness has been proposed as a unifying driver of invasion, treatment resistance, and relapse in oral squamous cell carcinoma (OSCC). We synthesized two complementary evidence streams to determine whether higher stemness predicts poorer survival in OSCC: (i) computational stemness signatures derived from transcriptomic/epigenetic data and (ii) tissue cancer stem cell (CSC) immunophenotypes by immunohistochemistry (IHC). **Methods:** Following PRISMA 2020, we searched PubMed/MEDLINE, Embase, Scopus, and SciELO. Adults with histologically confirmed OSCC were eligible. Primary outcome was overall survival (OS); disease-specific survival (DSS) and recurrence-free survival (RFS) were secondary. Two parallel meta-analyses pooled effects within domains; random-effects restricted maximum likelihood (REML) models were applied. **Results:** Of 785 records, 11 studies met criteria. For computational signatures (k = 6), higher stemness was associated with poorer OS (pooled HR 2.24, 95% CI 1.61–3.12; I^2^ ≈ 49%). Sensitivity excluding the single unadjusted Kaplan–Meier (KM)-derived estimate yielded a similar effect (HR 2.13, 95% CI 1.56–2.89). For CSC-IHC (main analysis, k = 2), CSC-positive profiles predicted worse OS (pooled HR 2.01, 95% CI 1.42–2.84; I^2^ ≈ 0%); results were robust to excluding an internally inconsistent study (single-study HR 2.078). An exploratory sensitivity analysis, including a 1-year HR (different time horizon), increased heterogeneity and was not considered definitive. A functional meta-synthesis converged on epithelial–mesenchymal transition/extracellular matrix remodeling, hypoxia/glycolysis, redox/ferroptosis resistance, and ribosome/rRNA biogenesis, supporting biological plausibility across modalities. **Conclusions:** Across computational and IHC evidence, stemness consistently portends inferior OS in OSCC, offering a biologically anchored framework for risk stratification and testable therapeutic hypotheses.

## 1. Introduction

Oral squamous cell carcinoma (OSCC) accounts for the vast majority of malignancies of the oral cavity and remains a major global health burden. According to recent global estimates, hundreds of thousands of new cases are diagnosed annually, and mortality remains substantial, with persistent disparities across regions and health systems [1]. Despite notable advances in surgery, radiotherapy, systemic therapy, and combined multimodal regimens, the 5-year overall survival (OS) of OSCC has plateaued at around 50–60% in many cohorts, largely due to loco-regional relapse, cervical lymph node metastasis, and treatment resistance [1,2]. Understanding—and ultimately targeting—the biological programs that fuel invasion, metastasis, and therapeutic failure is therefore a priority in OSCC [3].

A central paradigm that has reshaped our view of epithelial malignancies is the existence of intra-tumoral heterogeneity and plasticity. Single-cell transcriptomic mapping in head and neck squamous cell carcinoma (HNSCC) has revealed diverse malignant cell states—including partial epithelial-to-mesenchymal transition (p-EMT) and stress-responsive programs—that vary across tumor niches and associate with invasion and immune evasion [4]. Within this landscape, a subpopulation of tumor cells with stem-like properties—operationally defined as cancer stem cells (CSCs)—is thought to sustain tumorigenesis and relapse. The functional existence of CSCs in HNSCC was established by classical experiments demonstrating that CD44-positive fractions possess enhanced self-renewal, tumorigenicity, and differentiation capacity [5].

Over the last decade, two broad evidence streams have matured in parallel. The first stream is clinicopathologic and immunohistochemical (IHC), quantifying CSC phenotypes in patient tissues. Early OSCC work showed that the CD44+/CD133+ immunophenotype correlated with matrix metalloproteinase-9 (MMP-9) activity and adverse outcomes in early-stage disease [6]. In a large single-center cohort (n ≈ 231), high expression of the cystine–glutamate antiporter *SLC7A11*/xCT—alone or in combination with CD44—was independently associated with worse recurrence-free survival (RFS), disease-specific survival (DSS), and OS [7]. More recently, spatially resolved profiling at the invasive tumor front (ITF) demonstrated that ALDH1high and p75NTRhigh status and, especially, a composite CSChigh/E-cadherinlow profile were independent predictors of metastasis and poorer survival, suggesting that CSC-linked biology at the ITF captures clinically relevant aggressiveness [8].

The second stream is molecular-computational, which derives ‘stemness’ or stemness-related risk scores from bulk or single-cell omics. The mRNA expression-based stemness index (mRNAsi), trained on pluripotent stem cells and their differentiated progeny, provides a quantitative measure of oncogenic de-differentiation and has been widely applied across cancers [9]. In OSCC specifically, an mRNAsi-associated, multi-gene prognostic signature has been developed and validated; high mRNAsi and the derived risk score were linked to inferior survival and to distinct features of the tumor microenvironment [10]. Independent groups have constructed additional OSCC risk models using lncRNA panels [11], single-gene/lncRNA candidates with functional validation (e.g., *HOTAIRM1*) [12], and, most recently, CSC-gene-derived signatures anchored in single-cell datasets and validated across external cohorts, with supportive in vitro experiments [13]. These data complement earlier OSCC genomic and epigenetic signatures, such as a 13-gene HPV-negative OSCC signature with external validation [14] and a 7-CpG methylation classifier with prognostic capacity [15].

Despite consistent signals from both streams, they have seldom been brought together in an integrative, quantitative synthesis tailored to OSCC. IHC studies differ in antibodies, scoring systems, and cut-points; endpoints vary (OS, DSS, RFS); and cohort mix (stage, treatment, HPV status) and covariate adjustment are heterogeneous [6,7,8]. Molecular-computational models likewise differ in feature selection, normalization, training/validation splits, and reporting of calibration or decision-curve analyses [7,12,13]. As a result, clinicians and translational researchers lack a clear, comparative understanding of how phenotypic CSC markers relate to algorithmic stemness indices in terms of prognostic performance, clinical utility, and underlying biology in OSCC. Moreover, the degree to which these modalities converge on shared malignant programs—such as de-differentiation, EMT, metabolic rewiring, or hypoxia signaling—has not been systematically mapped in OSCC.

Accordingly, a systematic review and meta-analysis is proposed to examine two complementary evidence streams—immunohistochemistry-defined markers and transcriptomic/epigenetic indices of stemness. Therefore, the review aims to critically appraise and, where appropriate, synthesize time-to-event evidence, and to explore, if feasible, a functional integration of gene features that could illuminate recurrent biological programs underlying poor prognosis [1,4,5,6].

We aim to compare the prognostic performance of computational stemness signatures and IHC-defined CSC markers in OSCC with respect to overall survival, and—when reported—disease-specific and recurrence-free survival, and to explore, where feasible, a functional synthesis of gene features to reveal shared biological programs relevant to prognosis.

## 2. Materials and Methods

### 2.1. Reporting Standards and Registration

This systematic review and meta-analysis was designed and reported in accordance with PRISMA 2020 [16]. The protocol was developed a priori, aligned with best practices for prognostic biomarker research, and registered in PROSPERO (CRD420251158151).

### 2.2. Eligibility Criteria

Eligibility was defined a priori using the PICOS framework:

- Participants (P): Adults with histologically confirmed oral squamous cell carcinoma (OSCC), irrespective of stage, primary treatment, or care setting.
- Interventions/Exposures (I): Two exposure families were considered: (i) computational stemness models derived from omic data (e.g., mRNAsi/OCLR-based indices, lncRNA or multigene prognostic signatures, and CSC gene-anchored models); and (ii) cancer stem cell immunomarkers assessed by immunohistochemistry (e.g., CD44, ALDH1/ALDH1A1, CD133, CD24, *SLC7A11*/xCT, including composite or invasive tumor front panels).
- Comparators (C): High versus low stemness risk or biomarker expression according to study-defined thresholds (e.g., median split, prespecified score, validated cut-off).
- Outcomes (O): Time-to-event outcomes with extractable effect estimates: overall survival (primary), and disease-specific and/or recurrence-free survival (secondary), preferentially reported as hazard ratios (HRs) with 95% confidence intervals. Clinicopathologic associations were collected when available.
- Study design (S): Human cohort studies (retrospective or prospective), including analyses of public OSCC datasets that report or allow derivation of HRs.

Studies meeting the PICO but lacking an extractable hazard ratio and 95% confidence interval (and without sufficient information for reliable estimation) were eligible for qualitative synthesis only and were not included in quantitative meta-analysis.

We excluded non-OSCC head-and-neck cancers when OSCC data were not separable, animal or in vitro research, reviews and editorials, conference abstracts without extractable data, and studies lacking time-to-event metrics or sufficient information to estimate HRs. When overlapping cohorts were identified, the most comprehensive or recent report with unique participants was retained. For the CSC domain, tissue-level immunohistochemistry was required; non-IHC protein assays were not eligible.

### 2.3. Information Sources and Search Strategy

We searched PubMed/MEDLINE, Embase, Scopus, and SciELO from inception to September 2025 without language restrictions. We augmented database searches with backward citation screening, forward citation tracking, and author contact for clarification or missing survival estimates. The strategies combined controlled vocabulary (MeSH/Emtree) and free-text terms for OSCC, stemness/CSC biology, and survival endpoints. To minimize retrieval bias, we performed a limited Google Scholar search (first ~100 records by relevance; patents and “citation-only” items excluded) strictly to identify peer-reviewed articles that might not have been captured by database indexing and to trigger citation chasing.

### 2.4. Selection Process

Two reviewers independently screened titles and abstracts, followed by full-text assessment against eligibility criteria. Disagreements were resolved by discussion or adjudication by a third reviewer. Reasons for full-text exclusion were documented, and the study flow was summarized in a PRISMA diagram.

### 2.5. Data Collection and Items

Two reviewers independently extracted data using a piloted form. Extracted items included bibliographic information; country and setting; design; sample size and demographics; tumor characteristics (site, stage, HPV status when available) and treatment context; follow-up; exposure details; outcome definitions; and effect estimates. For the computational domain, we recorded data source (TCGA/GEO/ICGC or single-cell-anchored discovery), feature set (mRNAsi/lncRNA/CSC-gene), modeling approach (e.g., LASSO/OCLR/Cox), score derivation and cut-off, and validation strategy (internal/external). For the CSC-IHC domain, we captured the marker(s), antibody and scoring method, cut-off definition, and anatomical compartment (tumor center versus invasive front). When both univariable and multivariable HRs were available, multivariable estimates were preferred. Disagreements were reconciled by consensus or author contact.

For studies that did not report HRs directly, HRs were reconstructed from Kaplan–Meier (KM) survival curves using a standardized protocol. This included (i) digital extraction of survival probabilities using DigitizeIt (version 2.6.9); (ii) estimation of event counts and time-to-event distributions; and (iii) computation of log(HR) and its standard error using the Parmar–Tierney framework. Landmark survival probabilities, numbers at risk (when reported), and censoring patterns were incorporated to maximize precision. When both adjusted and unadjusted estimates were available, only adjusted HRs were used. KM-derived HRs were included solely when no alternative estimate was provided.

### 2.6. Risk of Bias Assessment

Risk of bias was assessed with the QUIPS tool across six domains (study participation, attrition, prognostic factor measurement, outcome measurement, confounding, and statistical analysis/reporting) [17]. Reporting quality was appraised against relevant REMARK items [18]. Two reviewers conducted assessments independently with consensus resolution; results were summarized in tables and figures.

### 2.7. Certainty of Evidence

Certainty was rated using GRADE adapted for prognostic evidence, considering risk of bias, inconsistency, indirectness, imprecision, and small-study/publication bias [19]. We classified certainty for each key outcome within each domain as high, moderate, low, or very low and provided explicit rationales.

### 2.8. Data Synthesis and Statistical Analysis

Within a unified stemness framework, two parallel quantitative syntheses were prespecified: (i) computational stemness signatures (e.g., mRNAsi-, lncRNA-, or CSC-gene-derived models) and (ii) CSC immunomarkers by immunohistochemistry (e.g., CD44, ALDH1/ALDH1A1, CD133, *SLC7A11*/xCT, composite invasive-front panels). Because measurement properties and bias structures differ between modalities, effects were pooled within, but not across, domains. The primary effect measure was the log hazard ratio for overall survival; disease-specific and recurrence-free survival were analyzed when reported. Random-effects models with restricted maximum likelihood (REML) were used. For studies reporting multiple independent cohorts, we first combined within-study effects via fixed-effect methods to derive a single study-level estimate. Where only continuous risk-per-unit HRs were available for computational scores, we synthesized them using the generic inverse-variance approach; when comparable high–low contrasts existed, those were preferred for interpretability.

Between-study heterogeneity was quantified using I^2^ and τ^2^. Influence diagnostics and leave-one-out analyses were applied to identify outliers and influential studies. Potential small-study effects were examined through funnel plots and Egger’s test when ≥10 studies were available for an outcome within a domain. Two-sided *p*-values < 0.05 were considered statistically significant, and 95% prediction intervals were reported where feasible.

Where quantitative pooling was not appropriate due to heterogeneity or insufficient data, a structured narrative synthesis summarized study characteristics and effect directions/magnitudes, supported by comparative tables and figures.

We prioritized directly reported HRs with 95% confidence intervals (CIs). If unavailable and assumptions were defensible and reproducible, we considered estimating HRs from Kaplan–Meier curves using validated approaches; otherwise, we contacted study authors. Studies for which an HR/CI could not be obtained or estimated reproducibly were included in the qualitative synthesis only and were not entered into meta-analyses.

To prevent double-counting of overlapping datasets, especially from The Cancer Genome Atlas (TCGA) and the International Cancer Genome Consortium (ICGC), each computational study was screened for primary data sources. When multiple studies reanalyzed the same parent dataset (e.g., TCGA-OSCC), only one HR estimate per cohort was included in the quantitative synthesis. Studies deriving signatures from the same dataset but reporting overlapping or redundant prognostic HRs were treated as non-independent; only the most adjusted or methodologically rigorous HR was retained. This procedure ensured that no dataset contributed more than once to any pooled effect.

### 2.9. Subgroup Analyses and Meta-Regression

Subject to data sufficiency, exploratory subgroup analyses or meta-regressions considered modifiers such as platform (RNA-seq vs. microarray), modeling approach (LASSO vs. One-Class Logistic Regression vs. other), validation (internal vs. external), and data source (TCGA vs. others) for the computational domain; and marker type, compartment (center vs. invasive front), antibody/scoring method, and cut-off definition for the CSC-IHC domain. Clinical factors (stage distribution, HPV status if reported, primary treatment, and geography) were also explored. Any inter-domain comparisons were strictly narrative and exploratory.

### 2.10. Sensitivity Analyses

Sensitivity analyses included restriction to studies at low or moderate risk of bias, preference for multivariable HRs, exclusion of outliers or highly influential studies, and testing alternative random-effects estimators to assess robustness of pooled effects.

Analyses were planned in Python (v3.11) and/or Review Manager (RevMan, v5.4). Forest and funnel plots were generated using the corresponding libraries.

### 2.11. Functional Meta-Synthesis

For computational signatures providing gene lists, gene symbols were harmonized according to HGNC nomenclature, duplicates were removed, and over-representation analyses (e.g., GO, KEGG) and protein–protein interaction mapping were performed to identify convergent pathways. These exploratory analyses served to contextualize—but not to replace—the prognostic meta-analyses.

To generate the functional meta-synthesis gene set, all genes, lncRNAs, CpG-mapped loci, and pathway annotations reported in the computational signatures were extracted verbatim from each primary study. Gene identifiers were harmonized using HGNC-approved symbols and collapsed across signatures to avoid redundancy. Each element was mapped to biological pathways using curated resources, including KEGG, Reactome, Gene Ontology, and MSigDB. Recurrent functional themes (e.g., EMT/ECM remodeling, hypoxia/glycolysis, redox/ferroptosis, ribosome/transcriptional programs, and therapy resistance) were identified through frequency analysis and independently verified by two reviewers. The resulting integrative set formed the basis for the updated functional network.

## 3. Results

### 3.1. Study Selection

A comprehensive search across PubMed/MEDLINE, Embase, SciELO, and Scopus yielded 685 records. Google Scholar yielded no additional eligible records after de-duplication. After removing 340 duplicates, 326 unique records underwent title/abstract screening. Studies clearly outside the scope—such as reports on non-oral head-and-neck sites without separable OSCC data; in-vitro or animal-only studies; reviews, editorials, letters, or conference abstracts; preprints; imaging/radiomic reports without biomarker assessment; non-IHC protein assays for the CSC domain; and computational/omics studies lacking survival endpoints or hazard ratios—were excluded. Nineteen full-text articles were assessed for eligibility. The full database-specific search strategies are provided in Supplementary Table S1. At full-text review, eight articles were excluded for predefined reasons (non-OSCC or non-separable HNSCC cohorts; absence of time-to-event outcomes or insufficient data to derive HR/CI; computational models without survival reporting or not reproducible). Supplementary Table S2 lists the eight full-text articles excluded at the eligibility stage, together with explicit reasons for their exclusion.

**Figure 1:**
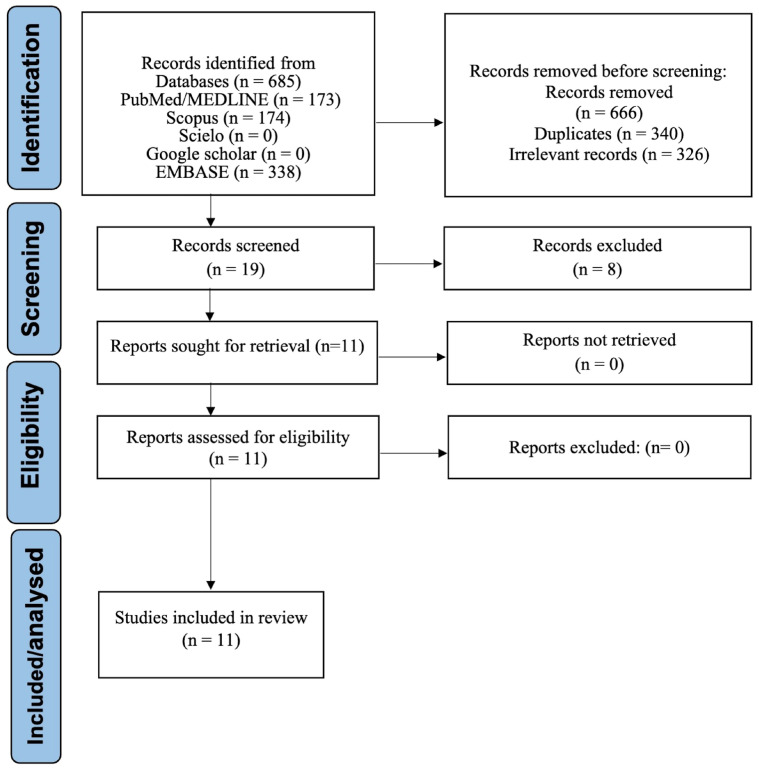
PRISMA 2020 flow diagram of study selection.

Eleven studies met the inclusion criteria; all were included in the qualitative synthesis, and nine contributed to the quantitative meta-analyses [6,7,8,10,11,12,13,14,15]. Two studies were synthesized narratively only [20,21]. The selection process is depicted in Figure 1.

### 3.2. Characteristics of Included Studies

The eleven included studies comprised two complementary evidence streams: six developed or validated computational stemness signatures or indices using transcriptomic/epigenetic data [10,11,12,13,14,15], and three evaluated CSC immunophenotypes in tissue by IHC [6,7,8]; two additional IHC reports were eligible only for narrative synthesis [20,21]. Overall survival (OS) was the primary endpoint across studies, with disease-specific survival (DSS) and recurrence-free survival (RFS) reported in a subset [7]. Most computational studies reported multivariable Cox models and HRs with 95% confidence intervals; IHC studies typically adjusted for clinicopathologic covariates and specified marker cut-offs. Table 1 summarizes design features, exposures, endpoints, and effect-estimate availability for each study.

### 3.3. Computational Prognostic Signatures (Primary Outcome, Overall Survival)

**Figure 2:**
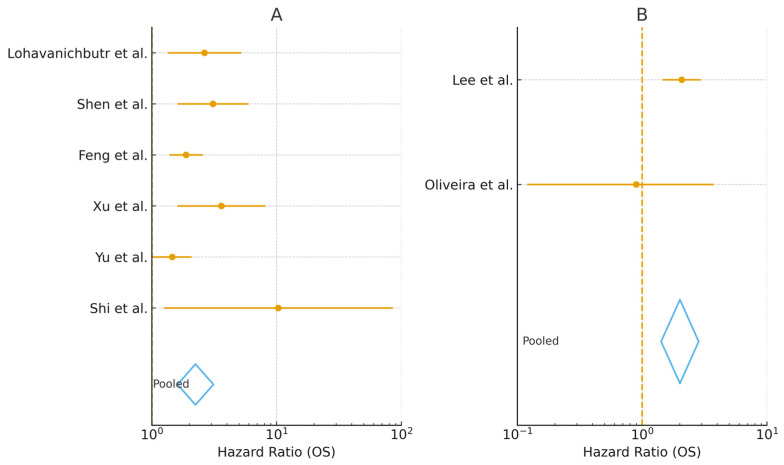
Forest plot of prognostic effects on overall survival (single figure with two halves): (**A**) (left): computational stemness signatures (k = 6) [10,11,12,13,14,15]. (**B**) (right): CSC immunohistochemistry (k = 2, main analysis) [6,7]. Points = study HRs with 95% CIs; diamonds = random-effects pooled estimates.

Six studies contributed signature-level hazard ratios for overall survival [10,11,12,13,14,15]. Random-effects pooling showed that high stemness/risk was associated with worse survival (pooled HR 2.24, 95% CI 1.61–3.12; I^2^ 49.1%). A sensitivity analysis excluding Shi et al. [13]—which contributed a KM-derived, unadjusted signature-level HR of 10.32 (95% CI 1.25–85.25) in the ICGC cohort—yielded a similar estimate (pooled HR 2.13, 95% CI 1.56–2.89; I^2^ 46.4%). Study-level effect sizes are provided in Supplementary Table S3, and the dual-panel forest is shown in Figure 2 (panel A).

#### 3.3.1. Stemness-Related Gene Features Identified Across Computational Signatures

Across the six computational studies included, a set of recurrent stemness-related gene features was identified. The stemness signatures incorporated genes involved in glycolytic activity, extracellular matrix organization, hypoxia response, redox regulation, transcriptional output, and ribosomal biogenesis. The 6-gene prognostic signature reported by Shi et al. [13] included *PGK1*, *P4HA1*, *ADM*, *PTGR1*, *RPL35A*, and *POLR1D*, each contributing to different components of cellular metabolism, structural remodeling, and biosynthetic machinery. The mRNAsi-related 11-gene signature described by Feng et al. [10] contained genes associated with metabolic adaptation and transcriptional programs linked to stemness.

Two studies developed long non-coding RNA-based models. Xu et al. [11] proposed a 14-lncRNA signature, in which several transcripts were involved in regulatory networks affecting immune activity, cell proliferation, and tumor microenvironmental signaling. Yu et al. [12] identified HOTAIRM1 as an individual lncRNA associated with higher stemness-related risk. These lncRNA signatures were derived from transcriptomic profiling and validated using multivariable time-to-event analyses.

Earlier gene-expression-based models also contributed stemness-associated features. The 13-gene signature developed for HPV-negative OSCC included genes linked to invasive and proliferative tumor states, while the 7-CpG methylation-based classifier captured epigenetic alterations connected to oncogenic de-differentiation. Together, these computational signatures collectively represented gene sets enriched in metabolic stress responses, transcriptional regulation, and structural or biosynthetic pathways typically associated with stemness in OSCC.

An integrative summary of all stemness-related markers, their associated pathways, and biological functions across computational and IHC evidence is provided in Table 2.

#### 3.3.2. Additional Stemness-Related Features from Multigene and Epigenetic Signatures

Beyond the 6-gene prognostic signature, several additional signatures reported stemness-associated gene sets relevant to OSCC. The mRNAsi-related 11-gene signature described by Feng et al. [10] included genes contributing to metabolic adaptation, transcriptional regulation, and cellular stress responses, although individual gene-level functional annotations were not fully detailed. The study by Yu et al. [12] identified HOTAIRM1 as a stemness-related transcript, and their associated gene-level analyses included expression components linked to differentiation and proliferative programs.

The 13-gene signature developed by Lohavanichbutr et al. [14] for HPV-negative OSCC incorporated genes associated with proliferative activity, extracellular matrix organization, and invasive tumor phenotypes. Meanwhile, the 7-CpG methylation classifier from Shen et al. [15] captured epigenetic modifications mapped to genes implicated in transcriptional control, cell-cycle processes, and tumor differentiation states. Together, these multigene and methylation-based signatures expand the spectrum of molecular features contributing to stemness-associated prognostic profiles in OSCC. All corresponding gene sets are included in Table 2 for comparative reference.

Although several of the 14 lncRNAs described by Xu et al. [11] participate in immune-related regulatory pathways, the original study conceptualized this panel as a stemness-associated prognostic signature derived from transcriptomic data.

### 3.4. CSC-IHC (Primary Outcome, Overall Survival)

Two studies provided extractable multivariable HRs and were meta-analyzed [6,7]. The pooled effect indicated poorer survival in CSC-positive phenotypes (pooled HR 2.01, 95% CI 1.42–2.84; k = 2). Notably, Oliveira et al. [6] reported Cox results as “RR” with an internally inconsistent confidence interval and *p* value (RR 0.897, 95% CI 0.120–3.746; *p* = 0.027); therefore, this study was retained in the main pool with caution and explicitly flagged in Table 1. A sensitivity analysis excluding Oliveira et al. [6] left a single eligible study [7] with HR 2.078 (95% CI 1.457–2.962). Including Ortiz et al. [8] (ALDH1 at the invasive tumor front) as a 1-year hazard ratio in an exploratory sensitivity analysis markedly increased heterogeneity (pooled HR 0.77, 95% CI 0.16–3.81; I^2^ 73.7%), supporting its exclusion from the primary IHC meta-analysis due to the different time horizon. See Figure 2 (panel B) for the forest plot and Table 3 for pooled estimates and sensitivity scenarios.

Two IHC reports provided survival comparisons but lacked extractable Cox HRs [20,21]. Both studies supported the direction of association between CSC-related immunophenotypes and adverse outcomes, but were not pooled quantitatively.

Given that only two studies contributed to the primary IHC meta-analysis, the pooled estimate should be regarded as technically limited. The internal inconsistency between the hazard ratio and *p*-value reported by Oliveira et al. [6] further reduces the reliability of that individual estimate and therefore affects the stability of the pooled HR. In addition, there was substantial methodological heterogeneity across IHC studies—including differences in antibody selection, staining procedures, scoring algorithms, cut-off definitions, and tumor regions assessed—which constrains the robustness of the quantitative findings.

#### Stemness-Related Immunohistochemical Markers Identified Across Included Studies

The three immunohistochemical studies meeting the inclusion criteria reported several cancer stem cell (CSC)-related markers examined in oral squamous cell carcinoma tissues. The markers assessed across these studies included surface adhesion molecules, aldehyde-metabolizing enzymes, cystine transporters, and epithelial cell–cell adhesion proteins.

Oliveira et al. [6] evaluated the combined CD44+/CD133+ phenotype. CD44 is a cell-surface glycoprotein associated with adhesion and migration, while CD133 is a pentaspan transmembrane protein commonly used to identify stem-like subsets within epithelial tumors. Lee et al. [7] examined CD44 together with SLC7A11/xCT, a component of the cystine–glutamate transporter system, which is involved in redox regulation. Their immunohistochemical analysis quantified expression levels of both markers within tumor tissues.

Ortiz et al. [8] assessed stemness features at the invasive tumor front, primarily focusing on ALDH1, an aldehyde dehydrogenase associated with cellular detoxification processes, and E-cadherin, a transmembrane protein involved in epithelial cell–cell adhesion. The authors also reported a composite pattern combining high CSC-related marker expression with reduced epithelial adhesion.

Together, these IHC studies evaluated marker profiles that included adhesion-related molecules (CD44, E-cadherin), aldehyde metabolism enzymes (ALDH1), transmembrane stemness-associated proteins (CD133), and redox-associated transporters (SLC7A11/xCT), providing tissue-level assessment of stemness-related phenotypes in OSCC.

### 3.5. Functional Meta-Synthesis

**Figure 3:**
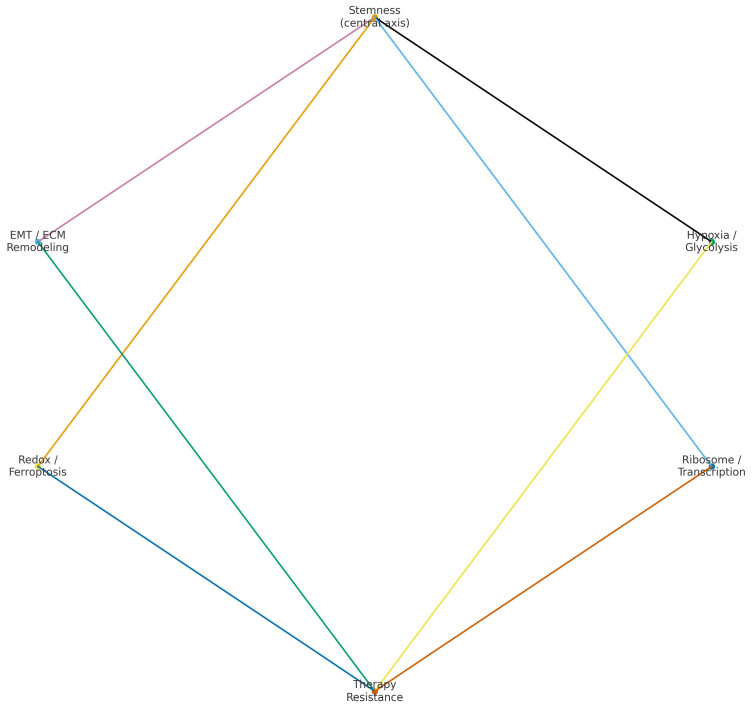
Functional meta-synthesis network illustrating the major stemness-related biological programs identified across computational and immunohistochemical studies. Each labeled domain (EMT/ECM remodeling, hypoxia/glycolysis, redox/ferroptosis, ribosome/transcription, and therapy resistance) represents a recurrent pathway cluster. Colored connecting lines depict conceptual relationships among these biological domains based on co-occurrence patterns in the included gene signatures. The layout reflects an abstracted, integrative overview rather than a mechanistic pathway map. Network visualization was generated using R igraph with force-directed layout optimization.

Mapping computational gene features and CSC immunophenotypes onto biological programs revealed convergent themes plausibly underlying poor outcomes in OSCC. The 6-GPS highlighted glycolysis (*PGK1*), ECM remodeling/collagen maturation (*P4HA1*), hypoxia/angiogenic stress (*ADM*), eicosanoid/arachidonic-acid metabolism (*PTGR1*), and ribosome/rRNA biogenesis (*RPL35A*, *POLR1D*). IHC markers pointed to EMT/adhesion (*CD44*, reduced E-cadherin), redox resilience/ferroptosis resistance (*xCT*/*SLC7A11*), and aldehyde/retinoid detoxification (*ALDH1*). Taken together, these axes—EMT/ECM, glucose metabolism/hypoxia, ribosomal/transcriptional biogenesis, and oxidative-stress/ferroptosis—support a high-stemness phenotype with invasive plasticity and survival advantages. Figure 3 summarizes the distribution of stemness-related features across the major biological themes identified in the included studies, including EMT/ECM remodeling, hypoxia/glycolysis, redox/ferroptosis regulation, ribosomal/transcriptional programs, and therapy resistance.

Beyond the convergent programs identified across stemness-related signatures, several studies highlighted molecular features associated with therapy resistance. Markers such as SLC7A11/xCT, ALDH1, P4HA1, PGK1, and EMT-related profiles (including reduced E-cadherin) have been reported as contributors to resistance against oxidative damage, chemotherapeutic agents, or radiotherapy. These components, along with transcripts included in the lncRNA signatures [11], overlap functionally with redox regulation, metabolic adaptation, and microenvironmental remodeling.

### 3.6. Risk of Bias and Reporting Quality

**Figure 4:**
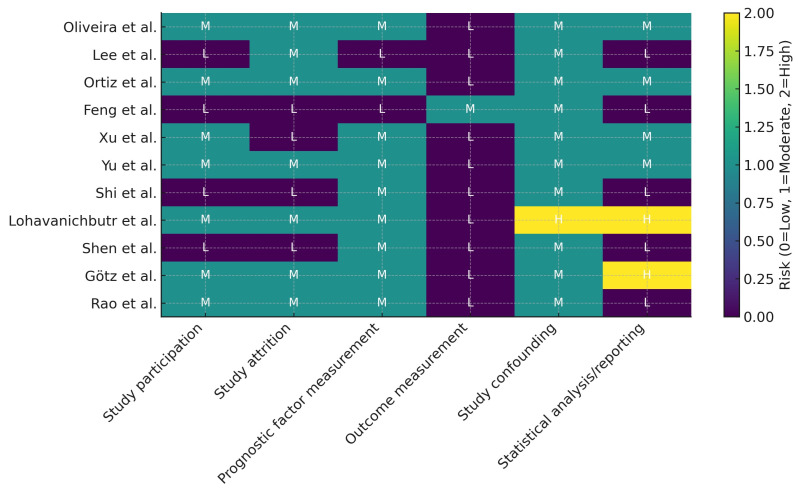
QUIPS risk-of-bias heatmap across six domains (study participation, study attrition, prognostic factor measurement, outcome measurement, study confounding, and statistical analysis/reporting) [6,7,8,10,11,12,13,14,15,20,21]. Each cell displays the risk level as L = Low, M = Moderate, or H = High, overlaid on a continuous color scale (0 = Low, 1 = Moderate, 2 = High) for visual emphasis.

Across the six QUIPS domains, most studies showed low risk for outcome measurement and moderate risk for study participation/attrition. Prognostic factor measurement was generally moderate—reflecting variability in IHC scoring (antibody, cut-offs, compartment) and in computational model construction. Confounding and statistical analysis/reporting ranged from low in multivariable, validated signatures [7,10,11,12,15] to moderate/high in studies with unadjusted or narrative-only survival comparisons [20,21]. The study-level QUIPS judgments are summarized in Figure 4. Adherence to key REMARK items was variable, with frequent gaps in cut-off justification, blinding of IHC assessors, and sample size rationale; these limitations informed our GRADE ratings.

### 3.7. Certainty of Evidence (GRADE)

Following GRADE for prognostic evidence [19], the certainty for the computational domain (OS) was moderate (downgraded for risk of bias and inconsistency; I^2^ ≈ 49%; some imprecision). For the IHC domain (OS), certainty was low (small k, clinical/measurement heterogeneity, and some imprecision; one study showed an internal CI–*p* inconsistency). For DSS/RFS, reporting was sparse, leading to low to very low certainty. Domain-specific ratings and rationales are presented in Table 4.

## 4. Discussion

This systematic review and meta-analysis integrated two complementary evidence streams to appraise the prognostic value of stemness in OSCC: (i) computational signatures derived from transcriptomic or epigenetic data, and (ii) tissue-level CSC immunophenotypes by IHC. Across 11 eligible studies, six contributed to the computational synthesis and three to the CSC–IHC synthesis, while two IHC reports were narratively summarized [6,7,8,10,11,12,13,14,15,20,21]. In the primary outcome, OS, computational models associated higher stemness with a materially increased hazard of death (pooled HR ≈ 2.2; Figure 2A; Table 3), and IHC markers likewise indicated poorer OS for CSC-positive profiles (pooled HR ≈ 2.0; Figure 2B; Table 3). The full set of stemness-related markers, their associated pathways, and their mapped biological functions is summarized in Table 2, which provides a consolidated view of the molecular and immunohistochemical features identified across studies. A functional meta-synthesis mapped the gene features and IHC markers onto convergent biological programs—EMT/ECM remodeling, hypoxia and metabolic reprogramming, redox resilience/ferroptosis resistance, and ribosome/rRNA biogenesis—supporting a coherent stemness-related pathobiology (Figure 3).

The computational literature spanned diverse approaches: mRNA-based stemness indices and multigene scores [10], lncRNA signatures [11,12], CSC-derived multigene panels [13], and earlier gene-expression models with external validation or HPV-specific stratification [14,15]. Despite heterogeneity in feature construction (OCLR/mRNAsi, LASSO-Cox, methylation-anchored models) and validation strategies, study-level effects consistently showed higher mortality for high-stemness groups. Notably, multivariable Cox modeling was common [10,11,12,15], strengthening causal interpretability by adjusting for clinicopathologic factors, whereas one study contributed an unadjusted KM-derived effect [13]; sensitivity analysis excluding this estimate yielded a similar pooled hazard (Table 3). These findings align with broader evidence in HNSCC and pan-cancer datasets: Malta et al. built the foundational OCLR-based stemness indices (mRNAsi/mDNAsi), showing that higher stemness tracks adverse outcomes and immune-checkpoint patterns across TCGA, including HNSCC [9]; Tian et al. derived an HNSCC eight-mRNAsi signature with robust discrimination (5-year AUC ≈ 0.77) and HPV-related differences, reinforcing prognostic relevance [22]; and Wu et al. integrated WGCNA with mRNAsi in HNSCC to identify ECM/collagen and PI3K–AKT-linked stemness genes, consistent with the biological themes captured by our six-gene panel and other signatures [23].

In addition to the 6-GPS signature, several earlier multigene and epigenetic models also contained markers relevant to stemness biology. The 11-gene mRNAsi-related signature [10] and HOTAIRM1-based model [12] included components linked to metabolic activity, transcriptional regulation, and proliferative states. The 13-gene HPV-negative OSCC signature [14] incorporated genes associated with extracellular matrix dynamics, cellular growth, and invasive potential. Similarly, the 7-CpG methylation classifier [15] reflected epigenetic patterns consistent with de-differentiated tumor states. Although these studies differed in design and depth of functional annotation, their gene sets align with the biological axes summarized in Table 2 and converge on the core stemness-related pathways highlighted in our functional meta-synthesis (Figure 3).

Several of the stemness-associated genes highlighted in the present synthesis have also been implicated in tumor progression and adverse outcomes in other solid malignancies. For instance, *PGK1* promotes glycolysis-driven aggressiveness in gastric and colorectal cancers; *P4HA1* regulates collagen remodeling and hypoxia adaptation in breast and lung cancers; and *SLC7A11/xCT* contributes to ferroptosis resistance in pancreatic, glioma, and prostate cancer models. These cross-tumor patterns reinforce the biological plausibility of the pathways identified in OSCC.

One computational model consisted of a 14-lncRNA signature in which several transcripts were annotated to immune-related pathways [11]. Despite this overlap, the authors of the original study explicitly framed the signature as representing stemness-associated risk, derived from de-differentiation-related transcriptional programs. Within this interpretative context, lncRNA-based models contribute to the broader stemness landscape by capturing regulatory layers that interact with both immune activity and cellular plasticity, consistent with our inclusion criteria.

A further dimension that emerged from the included studies relates to therapy resistance, which is widely recognized as a functional hallmark of stemness-driven tumor biology. Several markers identified in our review have been linked to increased resistance to chemoradiation or oxidative stress, including SLC7A11/xCT (ferroptosis avoidance), ALDH1 (detoxification capacity), PGK1 (glycolytic reprogramming associated with radioresistance), P4HA1 (hypoxia-mediated survival pathways), and EMT-associated profiles characterized by reduced E-cadherin expression. Although individual studies varied in the depth of mechanistic annotation, these elements converge on a phenotype with enhanced survival advantage under therapeutic pressure.

Across tissue studies, CD44 and xCT/*SLC7A11* overexpression was associated with inferior OS, and combined models (CD44 + xCT) showed particularly clear effects [7]. A phenotype defined by CSC enrichment and reduced E-cadherin—consistent with EMT and tumor-front aggressiveness—also marked poor prognosis in a short-term horizon [8]. One early-stage cohort reported a Cox estimate labeled as RR for CD44+/CD133+, with an internal CI–*p* inconsistency; we treated that study with caution and explored its influence in sensitivity analyses [6] (Figure 2B; Table 3). Overall, the IHC evidence—while smaller in k and more variable in cut-offs, antibodies, and anatomic compartment—converges on the same directional risk as the computational synthesis and is further supported by external OSCC literature: a meta-analysis found CD44 overexpression predicts worse OS and DFS in IHC-based cohorts [24]; loss of E-cadherin at the invasive tumor front correlates with poorer prognosis [25]; and strong ALDH1 immunoexpression identifies patients with adverse overall survival [26].

Integrating the gene features from computational signatures with the IHC-defined CSC biology yielded a parsimonious set of recurrent pathways (Figure 3). EMT/adhesion and ECM remodeling are supported by CD44 and reduced E-cadherin on the IHC side and by *P4HA1* on the transcriptomic side [7,8,13]; importantly, *P4HA1* acts as a hypoxia-surrogate and independently predicts poorer OS in OSCC, reinforcing this axis [27]. Metabolic stress programs are indicated by glycolysis (*PGK1*) and hypoxia (*ADM*), aligning with adverse OS in multiple reports [10,11,12,13] and with external evidence where *PGK1/P4HA1* co-define hypoxia-based prognostic signatures in OSCC [28]. Redox resilience and ferroptosis resistance are reflected by *xCT/SLC7A11* [7] and are mechanistically linked to glutathione-dependent suppression of lipid peroxidation, a targetable vulnerability in *SLC7A11*^high^ tumors [29]. Ribosome/rRNA biogenesis and transcriptional output (RPL35A, POLR1D) may sustain the biosynthetic capacity of aggressive phenotypes [13]. The recurrence of these signals across orthogonal measurement modalities strengthens the biological plausibility of a stemness–prognosis axis in OSCC.

The biological convergence observed across computational and IHC markers also carries translational relevance. Several pathways highlighted in the functional meta-synthesis align with therapeutic strategies currently under investigation. Inhibition of xCT/SLC7A11—the cystine/glutamate antiporter associated with ferroptosis resistance—is supported by drug-discovery efforts [30,31,32] and is being explored in early-phase clinical settings. Glycolytic regulators such as PGK1, which under hypoxia promote stem cell-like properties and EMT [33], intersect with trials of metabolic reprogramming agents in solid tumors. P4HA1-linked extracellular matrix remodeling and hypoxia adaptation [34,35] are targeted by compounds modulating collagen maturation or HIF-1 signalling, while ribosome/transcriptional programs associated with treatment resistance [36] correspond to emerging ribosome-directed therapies in head and neck oncology. Beyond therapeutics, computational stemness scoring may integrate into clinical workflows such as AI-assisted prognostic platforms currently being piloted in head and neck cancer. Together, these links illustrate how the identified pathways offer testable hypotheses for future translational and precision-oncology approaches in OSCC. These therapeutic connections explicitly highlight the clinical relevance of stemness-associated pathways in OSCC, addressing a major gap noted in prior stemness reviews.

QUIPS assessments (Figure 4) indicated low risk for outcome measurement in most studies, with moderate concerns in study participation/attrition and prognostic-factor measurement—chiefly reflecting scoring variability in IHC (antibodies, cut-offs, tumor compartment) and heterogeneity in signature construction. Confounding and analysis/reporting were generally low risk for multivariable computational studies [10,11,12,15], but higher in narrative-only IHC reports where no Cox HR was retrievable [20,21] and in the unadjusted estimate derived from KM curves [13]. These judgments informed model choices, sensitivity analyses, and the certainty ratings.

Using GRADE adapted for prognostic evidence, certainty for OS was judged moderate for computational signatures and low for IHC markers (Table 4). The computational domain was downgraded for risk of bias and inconsistency (I^2^ ≈ 49%) and mild imprecision; the IHC domain was limited by small k, measurement heterogeneity, and imprecision, with one study presenting an internal CI–*p* mismatch [6]. For DSS/RFS, reporting was sparse, yielding low to very low certainty. Importantly, directionality was concordant across domains, and computational findings were robust to exclusion of the unadjusted study [13] (Table 3).

Previous evidence syntheses examining prognostic markers in oral and head and neck squamous cell carcinoma have focused predominantly on tissue-level CSC immunophenotypes. In OSCC specifically, Tripathi et al. [37] conducted a meta-analysis restricted to immunohistochemical CSC markers—mainly CD44, CD24, CD133, and ALDH—and reported that CD133 expression was associated with poorer survival, whereas ALDH expression correlated with lymph node metastasis and advanced clinical stage. This study reinforced the adverse prognostic impact of selected CSC markers but did not include transcriptomic or epigenetic stemness signatures or perform any dual-modality synthesis.

At the broader head and neck level, Zhou and Sun [38] pooled data on the CSC marker ALDH1 in head and neck squamous cell carcinomas and found that ALDH1 positivity was associated with worse overall and disease-free survival, as well as with lymph node metastasis and poor differentiation. Likewise, Fan et al. [39] performed a meta-analysis of multiple CSC markers—including OCT4, SOX2, NANOG, ALDH1, and CD44—in HNSCC and showed that overexpression of stemness-related markers was linked to adverse survival outcomes. While these meta-analyses collectively support the prognostic relevance of CSC immunomarkers, they remain confined to protein-level expression assessed by immunohistochemistry and do not incorporate computational stemness indices or multi-gene models derived from large-scale omics datasets.

In contrast, the present review was designed around a dual-modality framework that integrates computational stemness signatures (derived from transcriptomic or epigenetic data) with CSC immunohistochemistry in OSCC. This approach allowed us to perform parallel meta-analyses within each domain and to compare the magnitude of prognostic effects under a unified stemness concept, rather than limiting the synthesis to individual protein markers. Furthermore, by conducting a functional meta-synthesis that maps the gene features from computational signatures onto convergent biological programs—such as epithelial–mesenchymal transition and extracellular matrix remodeling, hypoxia- and glycolysis-associated metabolic adaptation, redox and ferroptosis regulation, ribosome/rRNA biogenesis, and therapy resistance—our study provides mechanistic context that is not captured by CSC-only meta-analyses (Figure 3; Table 2).

Thus, while previous meta-analyses by Tripathi et al. [37], Zhou and Sun [38], and Fan et al. [39] robustly demonstrate that CSC immunomarkers are associated with worse outcomes, our dual-modality synthesis extends this evidence base by (i) integrating computational and tissue-based stemness measures within a single prognostic framework; (ii) directly comparing effect sizes and certainty of evidence across these modalities; and (iii) linking the identified signatures to biologically coherent, potentially targetable pathways that may inform translational strategies in precision OSCC management.

Taken together, this dual-modality approach offers an advance over prior CSC-only meta-analyses by positioning stemness as a unified prognostic axis supported by both transcriptomic models and tissue-level markers. This integrated perspective constitutes the key contribution of the present review.

This review has several strengths. First, it synthesizes two complementary evidence bases—computational and IHC—under a unified framework, pre-specifying parallel meta-analyses and a functional meta-synthesis to avoid cross-domain pooling. Second, the focus on time-to-event outcomes with HR/CI and preference for multivariable estimates enhances clinical interpretability. Third, predefined QUIPS, REMARK considerations, and GRADE provide a transparent appraisal of validity and certainty. Nonetheless, several limitations deserve emphasis. The number of eligible IHC studies with retrievable multivariable HRs was small (k ≤ 2 in the main pool), and measurement heterogeneity (antibody clone, immunostaining protocol, scoring criteria, cut-off thresholds, and anatomical compartment assessed) likely contributed to between-study variability. In addition, the quantitative synthesis of IHC studies should be interpreted with considerable caution. Only two studies met the criteria for the primary IHC meta-analysis, limiting statistical power and widening the uncertainty surrounding the pooled estimate. Notably, Oliveira et al. [6] presented an internal inconsistency between its reported hazard ratio and corresponding *p*-value, reducing confidence in that individual estimate and, by extension, introducing uncertainty into the pooled HR. Taken together, these factors indicate that the IHC-based conclusions remain exploratory and require validation in larger, methodologically standardized cohorts.

Among computational studies, diversity in feature engineering and validation strategies introduces model heterogeneity; one study relied on an unadjusted KM-derived effect [13]. Some outcomes (DSS/RFS) were insufficiently reported to allow robust pooling. Finally, the possibility of small-study effects could not be formally assessed (k < 10 per domain), and although publication bias cannot be tested statistically under these conditions, it remains a plausible limitation given the predominance of significant findings and the scarcity of neutral or negative reports.

The measurement heterogeneity identified in the QUIPS assessment—particularly across IHC protocols—highlights the need for greater methodological standardization in future biomarker research. Emerging technologies may substantially mitigate these limitations. Digital pathology platforms now enable reproducible, high-resolution quantification of protein expression using algorithmic scoring, thereby reducing inter-observer variability and subjectivity inherent to manual evaluation. AI-assisted image analysis can further standardize region-of-interest selection, segmentation of tumor versus stromal compartments, and continuous scoring of marker intensity. In parallel, harmonization of pre-analytical workflows (e.g., fixation time, antigen retrieval), antibody validation pipelines, and consensus-based cut-off definitions would improve comparability across studies. Collectively, these developments could reduce the risk of bias in future prognostic biomarker studies and increase the certainty of evidence under GRADE.

Taken together, these findings support the clinical relevance of stemness in OSCC [40,41]. In the near term, multigene computational scores may assist risk stratification—particularly when externally validated and adjusted for stage, nodal status, and treatment—to refine surveillance intensity or adjuvant decision-making [10,11,12,14,15]. On the tissue side, co-expression of CD44 with xCT/*SLC7A11* and reduction of E-cadherin at the invasive front identify patients with higher relapse risk who may benefit from closer follow-up or enrollment in biomarker-driven trials [7,8]. The functional map (Figure 4) points to testable therapeutic hypotheses, such as targeting cystine transport/redox buffering (xCT), collagen maturation (P4HA1), glycolysis (PGK1), or pathways coordinating stress adaptation (e.g., PI3K–AKT/TGF-β signaling highlighted across signatures) [7,10,11,12,13]. This strengthens the clinical interpretability of stemness as a prognostic axis and underscores its relevance for precision risk stratification in OSCC.

Future studies should prioritize the following: (i) prospective, treatment-annotated OSCC cohorts with pre-specified analysis plans and multivariable Cox modeling; (ii) harmonization of IHC protocols (antibody clones, scoring systems, and cut-offs) and explicit reporting by tumor compartment (center versus invasive front); (iii) external validation and net benefit evaluation (e.g., decision-curve analysis) for computational scores; (iv) joint modeling of CSC-IHC and computational features to test additive or synergistic prognostic value; and (v) mechanistic validation of targets suggested by the meta-synthesis (xCT, P4HA1, PGK1) using clinical–translational designs in OSCC. Preregistered protocols and REMARK-compliant reporting will further improve evidence quality.

Understanding stemness as a biological axis in oral squamous cell carcinoma provides a clinically useful framework for identifying high-risk patients. Computational stemness scores and cancer stem cell immunophenotypes—such as CD44 with xCT/*SLC7A11* expression—consistently predict poorer survival. Integrating these markers into pathology and risk assessment could refine prognostic evaluation, personalize surveillance intensity, and inform the design of biomarker-driven therapeutic strategies in precision oral oncology.

## 5. Conclusions

Across two complementary evidence streams, our review shows that “stemness” is a clinically relevant prognostic axis in oral squamous cell carcinoma. Computational signatures consistently associated higher stemness with about a two-fold increase in the hazard of death and retained magnitude in sensitivity analyses, yielding moderate certainty of evidence. Tissue CSC immunophenotypes (CD44 with xCT/*SLC7A11* and reduced E-cadherin at the invasive front) pointed in the same adverse direction, though with low certainty due to small k and measurement heterogeneity. A functional meta-synthesis integrated genes and markers into convergent programs—EMT/ECM remodeling, hypoxia and glycolysis, redox/ferroptosis resistance, and ribosome/rRNA biogenesis—providing biological plausibility for the observed prognostic signal.

Clinically, validated computational scores could augment risk stratification beyond standard clinicopathologic factors, while selected IHC patterns may help flag high-risk patients for intensified follow-up or biomarker-driven trials. Mechanistic and early-phase clinical studies targeting pathways highlighted here—such as xCT-mediated redox buffering, glycolysis (*PGK1*), collagen maturation (*P4HA1*), and PI3K–AKT/TGF-β signaling—are warranted. Collectively, this review delineates a coherent, biologically anchored framework for prognostic assessment in OSCC and defines practical benchmarks for the next generation of studies.

## Figures and Tables

**Table 1:** Characteristics of Included Studies.

Author, Year	Domain	Exposure/Signature	Endpoint(s) Reported	Effect Estimate (HR/CI)	Validation/Setting	Notes
Oliveira et al., 2014 [6]	CSC-IHC	CD44+/CD133+ phenotype	OS	RR (Cox) 0.897 (0.120–3.746) *	Tissue IHC	Reported as RR from a Cox model; CI and *p* are inconsistent (*p* = 0.027). Used with caution; flagged for verification.
Lee et al., 2018 [7]	CSC-IHC	CD44 and xCT (*SLC7A11*)	OS; RFS; DSS	HR 2.078 (1.457–2.962) (multivariable)	Tissue IHC	Center vs. invasive front reported; combined CD44 + xCT model.
Ortiz et al., 2024 [8]	CSC-IHC	CSChigh/E-cadlow; ALDH1high	OS (1-year HR); metastasis	HR 0.101 (0.0108–0.9355) at 1 year (multivariable)	Tissue IHC	Different time horizon; included only in sensitivity analyses.
Feng et al., 2021 [10]	Computational	mRNAsi index; 11-gene signature	OS	HR 1.883 (1.381–2.568) (multivariable)	Transcriptomics	Risk score model; Cox adjusted.
Xu et al., 2022 [11]	Computational	14-lncRNA signature	OS	HR 3.61 (1.60–8.12) (multivariable)	Transcriptomics	External validation reported.
Yu et al., 2022 [12]	Computational	*HOTAIRM1* (lncRNA)	OS	HR 1.458 (1.019–2.087) (multivariable)	Transcriptomics	Uni-/multivariable Cox reported.
Shi et al., 2024 [13]	Computational	6-gene signature (CSC-derived)	OS	HR 10.32 (1.25–85.25) (univariate, KM-derived)	Transcriptomics	Signature-level HR from KM panel; per-gene HRs also reported.
Lohavanichbutr et al., 2013 [14]	Computational	13-gene signature (HPV-negative)	OS	HR available	Transcriptomics	External validation reported.
Shen et al., 2017 [15]	Computational	7-CpG methylation signature	OS (AUC also reported)	HR available (combined validation)	Epigenetics + gene expression (GE)	External validation; combined in analysis.
Götz et al., 2018 [20]	CSC-IHC	ALDH1	OS (Cox mentioned; HR not reported/insufficient for extraction)	No Cox HR retrievable	Tissue IHC	Narrative only.
Rao et al., 2020 [21]	CSC-IHC	CSC panel	OS (Kaplan–Meier/log-rank only)	No Cox HR	Tissue IHC	Narrative only.

* Oliveira 2014 reports Cox results as “RR”. The CI (0.120–3.746) crosses the null while the *p* value is 0.027; the study is flagged for verification and interpreted with caution. CSC = cancer stem cell; IHC = immunohistochemistry; OS = overall survival; RFS = recurrence-free survival; DSS = disease-specific survival; HR = hazard ratio; CI = confidence interval; RR = relative risk (as labeled by the authors in a Cox model); Cox = Cox proportional hazards model; KM = Kaplan–Meier; AUC = area under the receiver-operating-characteristic curve; HPV = human papillomavirus; GE = gene expression; mRNAsi = mRNA-based stemness index; lncRNA = long non-coding RNA; E-cad = E-cadherin; xCT = *SLC7A11* transporter (cystine/glutamate antiporter); *HOTAIRM1* = HOXA transcript antisense RNA, myeloid-specific 1. Gene symbols follow HGNC conventions.

**Table 2:** Integrative summary of stemness-related markers, associated pathways, and biological functions identified across computational and immunohistochemical studies.

Marker/Gene/Signature	Evidence Stream	Associated Pathway	Biological Function	Stemness-Related Feature
PGK1	Computational	Glycolysis	Catalyzes ATP-generating step in glycolysis	Supports metabolic activity associated with stem-like states
P4HA1	Computational	ECM remodeling/Hypoxia	Collagen maturation and extracellular matrix organization	Reflects structural adaptation linked to stem-associated programs
ADM	Computational	Hypoxia/Angiogenesis	Peptide involved in hypoxia signaling and vascular response	Contributes to stress-responsive transcriptional programs
PTGR1	Computational	Eicosanoid/Arachidonic acid metabolism	Enzyme in prostaglandin metabolism	Associated with metabolic and inflammatory regulation
RPL35A	Computational	Ribosome biogenesis	Component of the 60S ribosomal subunit	Supports biosynthetic capacity typical of proliferative states
POLR1D	Computational	Transcription/rRNA synthesis	Subunit of RNA polymerase I	Contributes to enhanced transcriptional output
14-lncRNA signature	Computational	Regulatory non-coding networks	Modulation of gene expression and cellular signaling	Represents transcriptomic regulatory components associated with stemness patterns
HOTAIRM1	Computational	lncRNA-mediated regulation	Regulation of gene expression linked to differentiation pathways	Associated with high-risk stemness indices
Multigene signatures (11-gene, 13-gene)	Computational	Mixed (metabolic, structural, regulatory)	Combined gene sets influencing multiple cellular programs	Capture multi-pathway contributions to stem-associated tumor profiles
7-CpG methylation classifier	Computational	Epigenetic modification	DNA methylation affecting gene regulation	Reflects epigenetic patterns associated with de-differentiation
CD44	IHC	Adhesion/EMT	Cell-surface receptor for hyaluronan	Marks cell adhesion variability associated with CSC profiles
CD133	IHC	Stem cell membrane signaling	Pentaspan transmembrane protein	Used to identify subpopulations with stem-like properties
SLC7A11/xCT	IHC	Redox regulation/Cystine transport	Component of the cystine–glutamate antiporter	Contributes to regulation of redox balance in stem-associated phenotypes
ALDH1	IHC	Retinoid/Aldehyde metabolism	Detoxifying enzyme family	Reflects enzyme activity associated with cellular self-renewal
E-cadherin	IHC	Cell–cell adhesion	Maintenance of epithelial integrity	Altered expression observed in invasive tumor front phenotypes

**Table 3:** Summary of pooled meta-analyses and sensitivity scenarios.

Domain/Scenario	Studies	k	Model	Pooled HR (95% CI)	Heterogeneity (I^2^)	Notes
Computational—main	[10,11,12,13,14,15]	6	Random-effects (REML)	2.24 (1.61–3.12)	49.1%	Includes Shi et al. [13] (KM-derived)
Computational—sensitivity (excl. Shi et al. [13])	[10,11,12,13,14,15]	5	Random-effects (REML)	2.13 (1.56–2.89)	46.4%	Robust to exclusion
CSC-IHC—main	[6,7]	2	Random-effects (REML)	2.01 (1.42–2.84)	≈0%	Oliveira et al. [6] flagged; interpret with caution
CSC-IHC—sensitivity (exclude Oliveira et al. [6])	[7]	1	Fixed effect	2.078 (1.457–2.962)	Not applicable (k = 1)	Single-study estimate
CSC-IHC—sensitivity (include Ortiz et al. [8], 1-year)	[6,7,8]	3	Random-effects (REML)	0.77 (0.16–3.81)	73.7%	Different time horizon drives heterogeneity

CSC = cancer stem cell; IHC = immunohistochemistry; HR = hazard ratio; CI = confidence interval; REML = restricted maximum likelihood; Random-effects (RE) = model allowing between-study heterogeneity; Fixed-effect (FE) = single pooled effect assuming one true effect; I^2^ = inconsistency metric (%); k = number of studies; KM = Kaplan–Meier.

**Table 4:** Certainty of evidence (GRADE, adapted for prognostic questions).

Domain/Outcome	k (Studies)	Effect (Direction)	Overall Certainty	Downgrades (Why)	Notes
Computational—Overall survival (OS)	6	Higher stemness → ↑ mortality (pooled HR ≈ 2.2)	Moderate	Risk of bias (some unadjusted/variable modeling); Inconsistency (I^2^ ≈ 49%); Imprecision (wide CI in some studies)	Sensitivity excluding Shi et al. [13] similar; small-study effects not assessable (k < 10).
CSC-IHC—Overall survival (OS)	2 (main)	CSC-positive phenotype → ↑ mortality (pooled HR ≈ 2.0)	Low	Imprecision (wide CI, small k); Risk of bias (measurement/cut-off variation); Inconsistency (clinical heterogeneity)	Oliveira et al. [6] flagged (CI vs. *p* mismatch); Ortiz et al. [8] (1-year) used only in sensitivity.
Computational—DSS/RFS (if reported)	Sparse	Direction consistent where available	Very low	Serious imprecision; reporting sparsity; inconsistency likely	Not meta-analyzed due to limited reporting across studies.
CSC-IHC—DSS/RFS (subset)	Sparse	Direction consistent (worse in CSC-positive)	Low to very low	Serious imprecision; small k; variable definitions	Narrative emphasis; not pooled beyond OS.

CSC = cancer stem cell; IHC = immunohistochemistry; OS = overall survival; DSS = disease-specific survival; RFS = recurrence-free survival; HR = hazard ratio; CI/CIs = confidence interval(s); I^2^ = inconsistency metric (%); k = number of studies.

## Data Availability

The original contributions presented in this study are included in the article/Supplementary Material. Further inquiries can be directed to the corresponding author.

## Supplementary Materials

The following supporting information can be downloaded at: https://www.mdpi.com/article/10.3390/medsci14010021/s1, Table S1. Complete Search Strategies for Each Database. Table S2. Full-text articles excluded at the eligibility stage with reasons. Table S3. Study-level effect estimates and model attributes (by domain).
