# Supplementary material for: Computational Stemness and Cancer Stem Cell Markers in Oral Squamous Cell Carcinoma: A Systematic Review, Dual Meta-Analysis, and Functional Meta-Synthesis
Source: Med Sci (Basel). 2025 Dec 31;14(1):21. doi: 10.3390/medsci14010021 (PMC12821640; doi:10.3390/medsci14010021)
Supplement: Supplementary file 1 [file medsci-14-00021-s001.zip › Supplementary Table S2 Excluded Studies.pdf]

Supplementary Table S2. Excluded Full-Text Articles and Reasons for Exclusion

No.	Reference	Reason for Exclusion
1	Furudate K, Kasai S, Yoshizawa T, Sasaki Y, Fujikura K, Goto S, Ito R, Takagi K, Tanaka T, Kijima H, Kubota K, Itoh K, Kobayashi W, Takahashi K. Spatial colocalization and molecular crosstalk of myofibroblastic CAFs and tumor cells shape lymph node metastasis in oral squamous cell carcinoma. PLoS Genet. 2025 Sep 4;21(9):e1011791. doi:10.1371/journal.pgen.1011791.	Non-separable HNSCC cohort; lacks survival or hazard ratio data.
2	Zhuo S, Jing C, Liu X, Yue J, Zhang W, Zhang S. Daughter Cells Budding From PGCCs and Their Clinicopathological Significances in Oral Squamous Cell Carcinoma. J Oral Pathol Med. 2025 Sep;54(8):687-693. doi:10.1111/jop.70014.	Clinical correlations without time-to-event analysis or HR/CI reporting.
3	Qin TX, Zhu YY, Ng WH, Ng SK, Chek MF, Tang KD. Adipocytes promote cancer stemness properties in oral squamous cell carcinoma through C3/C3AR axis and sphingolipid metabolism. Cancer Lett. 2025 Sep 28;628:217848. doi:10.1016/j.canlet.2025.217848.	In vitro mechanistic co-culture study; no human cohort or survival data.
4	Kumar S, De T, Subramani J, Rangarajan A, Pal D. Combined analysis of somatic mutations and gene expression reveals nuclear speckles-associated enhanced stemness in gingivobuccal carcinoma under DNA damage response. Comput Biol Chem. 2025 Dec;119:108513. doi:10.1016/j.compbiolchem.2025.108513.	Transcriptomic cell line study; no patient-derived data or HR estimations.
5	Joshi P, Bane S, Chaturvedi P, Gera P, Waghmare SK. Establishment and characterization of patient-derived tongue squamous cell carcinoma cell lines. Hum Cell. 2025 May;38(4):102. doi:10.1007/s13577-025-01231-w.	In vitro cell line development; lacks clinical or survival outcomes.
6	Ilie IO, Camen A, Dumitrescu D, Munteanu MC, Matei M, Şerbănescu MS, Mărgăritescu C. Immunoprofile of some surface and cytoplasmic	Descriptive IHC study; no survival endpoints or HR reporting.

	peripheral cell adhesion molecules in oral squamous cell carcinoma. Rom J Morphol Embryol. 2025 Jan-Mar;66(1):179-197. doi:10.47162/RJME.66.1.17.	
7	Huang YH, Chien PJ, Wang WL, Hsu LS, Huang YM, Chang WW. Tribbles pseudokinase 3 drives cancer stemness in oral squamous cell carcinoma cells by supporting the expression levels of SOX2 and EGFR. Int J Mol Med. 2025 Mar;55(3):44. doi:10.3892/ijmm.2025.5485.	Functional in vitro study without clinical cohort or survival analysis.
8	Tripathi A, Singh M, Mishra P, Fatima N, Kumar V. Meta-Analysis of Prognostic Significance of Cancer Stem Cell Markers in Oral Squamous Cell Carcinoma. Asian Pac J Cancer Prev. 2024 Oct 1;25(10):3597-3607. doi:10.31557/APJCP.2024.25.10.3597.	Secondary meta-analysis; not a primary cohort study.
