# Supplementary material for: Computational Stemness and Cancer Stem Cell Markers in Oral Squamous Cell Carcinoma: A Systematic Review, Dual Meta-Analysis, and Functional Meta-Synthesis
Source: Med Sci (Basel). 2025 Dec 31;14(1):21. doi: 10.3390/medsci14010021 (PMC12821640; doi:10.3390/medsci14010021)
Supplement: Supplementary file 1 [file medsci-14-00021-s001.zip › Supplementary Table S1 SearchStrings.pdf]

Supplementary Table S1. Final database-specific search strategies

Database	Domain	Search string (copy/paste)
PubMed/MEDLINE	CSC by IHC	(("Mouth Neoplasms"[Mesh] OR oral[tiab] OR "oral cavity"[tiab] OR tongue[tiab] OR buccal[tiab] OR gingiv*[tiab] OR "floor of mouth"[tiab]) AND ("Carcinoma, Squamous Cell"[Mesh] OR "squamous cell carcinoma"[tiab] OR OSCC[tiab])) AND (CD44[tiab] OR ALDH1[tiab] OR "aldehyde dehydrogenase 1"[tiab] OR ALDH1A1[tiab] OR CD133[tiab] OR SLC7A11[tiab] OR xCT[tiab]) AND ("Immunohistochemistry"[Mesh] OR immunohistochemistry[tiab] OR IHC[tiab]) AND (surviv*[tiab] OR "hazard ratio"[tiab] OR cox[tiab] OR "Prognosis"[Mesh] OR "Survival Analysis"[Mesh]) NOT (larynx[tiab] OR laryngeal[tiab] OR pharyn*[tiab] OR oropharyn*[tiab] OR nasopharyn*[tiab])
PubMed/MEDLINE	Computational signatures/indices	(("Mouth Neoplasms"[Mesh] OR oral[tiab] OR "oral cavity"[tiab] OR tongue[tiab] OR buccal[tiab] OR gingiv*[tiab] OR "floor of mouth"[tiab]) AND ("Carcinoma, Squamous Cell"[Mesh] OR "squamous cell carcinoma"[tiab] OR OSCC[tiab])) AND (stemness[tiab] OR mRNAsi[tiab] OR OCLR[tiab] OR lncRNA[tiab] OR "gene signature"[tiab] OR "risk score"[tiab] OR "prognostic signature"[tiab]) AND ("Gene Expression Profiling"[Mesh] OR transcriptome[tiab] OR "RNA-Seq"[tiab] OR microarray[tiab] OR methylation[tiab]) AND (surviv*[tiab] OR "hazard ratio"[tiab] OR cox[tiab] OR "Prognosis"[Mesh] OR "Survival Analysis"[Mesh])

		NOT (larynx[tiab] OR laryngeal[tiab] OR pharyn*[tiab] OR oropharyn*[tiab] OR nasopharyn*[tiab])
Embase (Ovid)	CSC by IHC	((oral OR "oral cavity" OR mouth OR tongue OR buccal OR gingiv* OR "floor of mouth").ti,ab,kw.<br>AND ("squamous cell carcinoma".ti,ab,kw. OR oscc.ti,ab,kw. OR scc.ti,ab,kw.))<br>AND<br>(cd44.ti,ab,kw. OR aldh1.ti,ab,kw. OR cd133.ti,ab,kw. OR slc7a11.ti,ab,kw. OR xct.ti,ab,kw.)<br>AND<br>(surviv*.ti,ab,kw. OR "hazard ratio".ti,ab,kw. OR cox.ti,ab,kw.)
Embase (Ovid)	Computational signatures/indices	((oral OR "oral cavity" OR mouth OR tongue OR buccal OR gingiv* OR "floor of mouth").ti,ab,kw.<br>AND ("squamous cell carcinoma".ti,ab,kw. OR oscc.ti,ab,kw. OR scc.ti,ab,kw.))<br>AND<br>(stemness.ti,ab,kw. OR mrnasi.ti,ab,kw. OR oclr.ti,ab,kw. OR lncrna.ti,ab,kw. OR "gene signature".ti,ab,kw. OR "risk score".ti,ab,kw. OR "prognostic signature".ti,ab,kw.)<br>AND<br>(transcriptom*.ti,ab,kw. OR "rna-seq".ti,ab,kw. OR microarray.ti,ab,kw. OR methylation.ti,ab,kw. OR "gene expression".ti,ab,kw.)<br>AND<br>(surviv*.ti,ab,kw. OR "hazard ratio".ti,ab,kw. OR cox.ti,ab,kw.)
Scopus	CSC by IHC	TITLE-ABS-KEY(((oral W/3 (cavity OR tongue OR gingiv* OR floor OR buccal) W/3 (cancer* OR carcinoma* OR neoplasm* OR tumor*)) OR oscc) AND ("squamous cell carcinoma" OR scc))<br>AND TITLE-ABS-KEY((cd44 OR aldh1 OR "aldehyde dehydrogenase 1" OR aldh1a1 OR cd133 OR slc7a11 OR xct) AND (immunohistochemistry OR ihc))<br>AND TITLE-ABS-KEY(surviv* OR "hazard ratio" OR cox)<br>AND NOT TITLE-ABS-KEY(larynx OR laryngeal OR pharyn* OR oropharyn* OR nasopharyn*)

Scopus	Computational signatures/indices	TITLE-ABS-KEY(((oral W/3 (cavity OR tongue OR gingiv* OR floor OR buccal) W/3 (cancer* OR carcinoma* OR neoplasm* OR tumor*)) OR oscc) AND ("squamous cell carcinoma" OR scc))<br>AND TITLE-ABS-KEY((stemness OR mrnasi OR oclr OR lncrna OR "gene signature" OR "risk score" OR "prognostic signature") AND (transcriptome OR "RNA-Seq" OR microarray OR methylation))<br>AND TITLE-ABS-KEY(surviv* OR "hazard ratio" OR cox)<br>AND NOT TITLE-ABS-KEY(larynx OR laryngeal OR pharyn* OR oropharyn* OR nasopharyn*)
SciELO	IHC (ES)	("carcinoma epidermoide oral" OR "carcinoma escamocelular oral" OR "cáncer oral" OR "oral squamous cell carcinoma")<br>AND (CD44 OR ALDH1 OR CD133)<br>AND (superviv* OR "análisis de supervivencia" OR Cox OR "hazard ratio")
SciELO	IHC (PT)	("carcinoma espinocelular oral" OR "carcinoma de células escamosas oral" OR "câncer oral")<br>AND (CD44 OR ALDH1 OR CD133)<br>AND (sobreviv* OR "análise de sobrevivência" OR Cox OR "hazard ratio")
SciELO	Computational (ES/PT)	("oral squamous cell carcinoma" OR "cáncer oral" OR "câncer oral")<br>AND (stemness OR CSC OR "células madre del cáncer" OR "células-tronco do câncer" OR lncRNA OR mRNAsi)<br>AND (superviv* OR sobrevid* OR sobreviv* OR Cox OR "hazard ratio")
Google Scholar	Balanced (peer-reviewed only)	"oral squamous cell carcinoma" (stemness OR "cancer stem cell" OR CSC OR mRNAsi OR OCLR OR lncRNA OR CD44 OR ALDH1 OR CD133 OR SLC7A11 OR xCT) (survival OR "hazard ratio" OR Cox)
